# A biomimetic and xeno-free platform for corneal engineering: synergy between PRGF technology and human dental pulp stem cells

**DOI:** 10.3389/fbioe.2026.1877996

**Published:** 2026-07-09

**Authors:** Eduardo Anitua, Mar Zalduendo, María Troya, Iraia Reparaz, Mohammad H. Alkhraisat

**Affiliations:** 1 BTI-Biotechnology Institute, Vitoria, Spain; 2 UIRMI (UPV/EHU-Fundación Eduardo Anitua), University Institute for Regenerative Medicine & Oral Implantology, Vitoria, Spain; 3 Oral and Maxillofacial Surgery, Oral Medicine and Periodontics Department, Faculty of Dentistry, University of Jordan, Amman, Jordan

**Keywords:** bioengineered corneal substitute, human dental pulp stem cells, PRGF technology, scaffold-based corneal graft, tissue engineering

## Abstract

**Introduction:**

Even though the cornea is one of the most widely transplanted solid tissues, it is estimated that approximately 98% of patients requiring a transplant lack access to this sight-saving procedure. This critical gap is a direct consequence of a tremendous global donor shortage, highlighting the urgent need for bioengineered alternatives. Despite significant research efforts, creating a functional bioengineered cornea remains a major challenge. The present study investigates a novel biomimetic approach leveraging Plasma Rich in Growth Factors (PRGF) technology in combination with dental pulp stem cells.

**Material and methods:**

Human dental pulp stem cells (hDPSCs) were isolated, characterized, and differentiated into epithelial, stromal, and endothelial corneal phenotypes. Two distinct types of corneal grafts were developed, categorized by the differentiation stage of the incorporated hDPSCs. The bioengineered constructs consisted of a central bioactive PRGF-fibrin core embedding cells and sandwiched between a trilaminar anterior layer and a posterior cell sheet. The PRGF-derived supernatant was utilized as a medium supplement. The resulting corneal substitutes were evaluated for their optical and rheological properties, as well as cellular viability. Finally, the structural stability and remodeling kinetics of the bioengineered grafts were histologically examined and quantified.

**Results:**

Corneal grafts incorporating undifferentiated hDPSCs exhibited superior elasticity and transparency, while maintaining a higher cell density following the fabrication process. The more pronounced mass reduction observed in these constructs was concomitant with accelerated fibrin degradation and enhanced *de novo* collagen synthesis.

**Conclusion:**

Bioengineered grafts comprising a central PRGF membrane fibrin core integrated with hDPSCs to mimetically replicate the trilaminar cellular architecture of the cornea, represent a dynamic biomimetic candidate for future clinical applications in corneal transplantation surgery.

## Graphical Abstract

**Figure:**
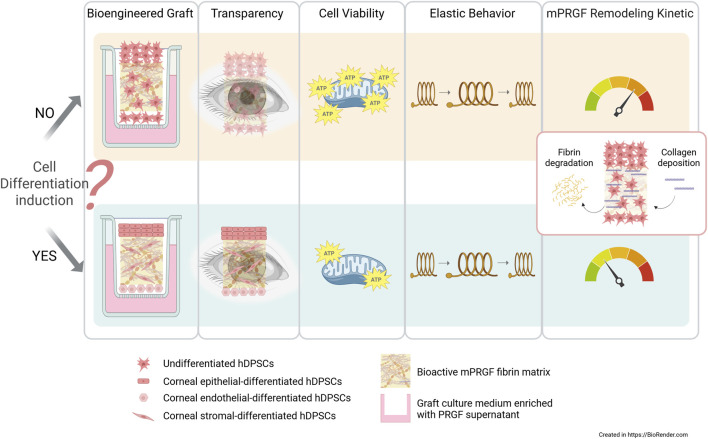

## 1 Introduction

The cornea is a specialized transparent tissue comprising three distinct layers: epithelium, stroma, and endothelium, from the outer to the inner surface, separated by two acellular interfaces referred to as Bowman’s layer and Descemet’s membrane (Espana and Birk, 2020; Raut et al., 2025). Its characteristic clarity is a direct result of the precise spatial organization of its cellular and extracellular components, its intrinsic avascularity, and the active metabolic homeostatic mechanisms maintained by the basal endothelial layer (Rates et al., 2022). The cornea is both mechanically strong and transparent, providing about 50% refractive power of the eye. However, genetic conditions involving abnormal deposits of materials in one or more layers of the cornea, along with corneal ectasias can lead to permanent visual impairment by compromising corneal curvature and transparency. Furthermore, severe bacterial, fungal, or viral keratitis, as well as chemical burns, and post-surgical complications often result in dense corneal scarring that obstructs the visual axis. Collectively, these pathologies represent a leading cause of global blindness, with keratoplasty frequently remaining the only viable option for vision restoration (Watson and Robaei, 2014). Despite the fact that the cornea is one of the most widely transplanted solid tissues (Zhou et al., 2023), it is estimated that approximately 98% of patients requiring a transplant lack access to this sight-saving procedure (Gain et al., 2016). This critical gap is a direct consequence of a tremendous global donor shortage, highlighting the urgent need for bioengineered alternatives.

Researchers have increasingly shifted their focus toward the development of alternatives to human donor corneas predicated on the necessity that an ideal graft must fulfill specific criteria related to corneal functionality, mechanical strength, biomimetic and reduced antigenic and inflammatory response from the recipient (Al-Shami et al., 2025). In an effort to generate viable corneal equivalents, a plethora of biomaterial systems and cellular sources have been used according to the tissue engineering principles (Marin-Tapia et al., 2025; Chi et al., 2023; Soroushzadeh et al., 2022; Javed and Daigavane, 2024). The primary methodologies, which have undergone significant evolution over the last decade, can be categorized into scaffold-free and scaffold-based strategies. Within the latter group, bioengineered systems are further classified into cellular or acellular constructs, depending on whether they incorporate regenerative cell populations or rely solely on a bioactive matrix to guide host tissue integration (Hussain and Pei, 2021). Cellular scaffold-free approaches such as cell injections, cell sheets and spheroids, enable the delivery of high cell densities to the target site. However, one crucial aspect for translational applications is ensuring cell retention, cell survival, and tissue integration. In this regard, Cell Sheet Technology enables the harvest of intact cellular layers that preserve cell contacts to the newly synthesized matrix without disrupting intercellular junctions, and exhibit bioadhesive properties that could facilitate sutureless integration (Lin et al., 2025; Anitua et al., 2024). Scaffold-free approaches would be more suitable for epithelium and endothelium reconstruction since these corneal layers do not contain complex matrix components. However, the difficult manageability of these cellular structures imposes limitations on their practical applications.

On the other hand, scaffold-based constructs provide the necessary structural template for tissue regeneration, a requirement that remains challenging to fulfil using scaffold-free methodologies. In this context, the degradation kinetics of the biomaterial represent a pivotal consideration; the scaffold must be progressively replaced by newly synthesized extracellular matrix during the remodeling process (Mayorca-Guiliani et al., 2025; Naba, 2024). A primary advantage of these approaches lies in their superior scalability and biomanufacturing potential, rendering them particularly effective for stromal regeneration and full-thickness corneal reconstruction. The possibility of combining diverse biomaterials, cell populations and signaling factors through different manufacturing techniques has promoted the development of numerous scaffold-based strategies (Ahearne et al., 2020; Azhar et al., 2026; Visalli et al., 2024; Tafti et al., 2022; Monostori et al., 2024; Orash et al., 2025). The mechanical and biological properties of these systems are governed by the intrinsic physicochemical characteristics of the biomaterial which are subsequently modulated by the cellular component through matrix active remodeling and paracrine signaling. To date, a variety of biomaterials have been explored in search of an optimal combination of optical, mechanical, and biological properties. In this context, natural tissues including the amniotic membrane, fish scales and decellularized corneal stroma have been extensively investigated (Lohchab et al., 2024; Akbaripour et al., 2026; Vijayaraghavalu et al., 2024). Furthermore, a wide repertoire of natural polymers such as collagen type I, gelatine, hyaluronic acid, silk protein, fibrin, alginate and chitosan, among others natural polymers, has been evaluated for their potential as corneal scaffold templates (Soleimani et al., 2023a; Wu et al., 2024; Li and Wang, 2025). Similarly, the properties of synthetic polymers (poly(dl-lactide-co-glycolide) (PLGA), polycaprolactone (PCL), polyethylene glycol (PEG), poly(methyl-methacrylate) (PMMA)) have garnered significant attention from researchers (Parekh et al., 2021; Wu et al., 2024). These are often utilized independently or as hybrid systems combined with natural polymers, as exemplified by the widely used gelatin methacrylate (GelMA) scaffold (Chai et al., 2026).

Regarding the cellular component of bioengineered grafts, corneal limbal, stromal and endothelial stem cells represent the most direct cell source for transplantation (Xiao et al., 2024; Wang et al., 2025). However, intensive research has shifted toward extraocular stem cell populations to circumvent the limitations of donor availability and autologous harvest. Researchers have explored a wide array of non-corneal stem cell alternatives from diverse niches, including the oral mucosa, bone marrow, amniotic membrane, adipose tissue, hair follicles, and dental pulp, among other origins, that possess the plasticity to differentiate into corneal phenotypes and repair damaged ocular tissue (Wang et al., 2023; Tang et al., 2025; Soleimani et al., 2023b).

Despite extensive research, the development of a bioengineered graft that comprehensively satisfies the stringent criteria for functional corneal substitution remains a significant challenge (Anitua et al., 2026). Individually, isolated biomaterials and specific cell populations exhibit inherent limitations that often impede full functional recovery. Consequently, a combinatorial strategy, integrating synergistic components from tissue engineering and regenerative medicine, is more likely to yield clinically successful outcomes by addressing the complex, multifactorial requirements of corneal restoration. In this context, Plasma Rich in Growth Factors (PRGF) Technology emerges as a versatile and valuable approach to overcome current limitations (Anitua et al., 2007; Orive and Anitua, 2021). The diverse range of PRGF formulations comprises a synergistic combination of human plasma-derived proteins and the complex secretome released by activated human platelets. The present research aims to evaluate a scaffold-based cellular strategy combining a bioactive PRGF fibrin membrane (Anitua et al., 2021) enriched with an array of regenerative signals, and a PRGF-derived supernatant applied to enrich the cell culture medium replacing the exogenous Fetal Bovine Serum (FBS) (Anitua et al., 2019). The remodeling process would be orchestrated by human dental pulp stem cells integrated into the three layers of the engineered graft.

## 2 Materials & methods

### 2.1 Dental pulp stem cell isolation and culture

The study was performed in accordance with the ethical standards from the Araba University Hospital Clinical Research Ethical Committee (BTIIMD-02-IV-24-COR) and conducted in accordance with the principles of the Declaration of Helsinki (2024 revision). Primary human dental pulp stem cells (hDPSCs) were isolated from one normal impacted wisdom tooth of a healthy donor (aged 14 years) following written parental informed consent. Isolation was achieved *via* the explant method. Briefly, tooth surface was disinfected and mechanically fractured at the cementum-enamel junction to retrieve the dental pulp. The tissue was minced into 1–2 mm^3^ fragments and cultured in Dulbecco’s modified eagle medium/Ham’s F-12 nutrient mixture (D-MEM/F-12) (1:1) (Gibco, Thermo Fisher Scientific Inc., Waltham, Massachusetts, USA) supplemented with 2 mM glutamine, 50 μg/mL gentamicin, 2.5 μg/mL amphotericin B and 10% FBS (all from Merck KGaA, Darmstadt, Alemania). Cultures were maintained under normal culture conditions (at 37 °C in a humidified 5% CO_2_ atmosphere), with medium changes every 2–3 days. Upon reaching 70%–80% confluence, cells growing out from the pulp explants were detached using an animal origin-free enzyme (TrypLE) (Gibco-Thermo Fisher Scientific Inc.) for sub-culturing or cryopreservation. Cell viability was confirmed *via* trypan blue dye exclusion. Primary cultures were then maintained with the isolation medium without amphotericin (onwards, routine culture medium). Experimental assays were performed using cells between the third and sixth passages to ensure phenotypic stability.

### 2.2 Analysis of the stemness nature of isolated hDPSCs

#### 2.2.1 Antigenic characterization

Stemness of hDPSCs was assessed by flow cytometry. Thus, the surface expression of CD73, CD90, CD14, CD19, CD45 was analyzed, according to the standard criteria established by the International Society for Cell and Gene Therapy (ISCT) (Dominici et al., 2006). Isotype-matched IgG1 and IgG2a antibodies conjugated to the corresponding fluorochromes were also included in the analysis. All reagents were obtained by Becton, Dickinson and Company (Franklin Lakes, NJ, USA). After washing and blocking, 2.5 x 10^5^ cells per sample were incubated with the antibodies for 1 h at 4 °C in the dark. Samples were then washed, fixed in 1% paraformaldehyde and analyzed using a Gallios flow cytometer (Beckman-Coulter, High Wycombe, Buckinghamshire, UK).

#### 2.2.2 Trilineage differentiation of hDPSCs

The ability of the isolated hPDLSCs to differentiate into cell phenotypes derived from the three embryonic germ layers was evaluated, to complete their characterization as stem cells. All the osteogenic, adipogenic and chondrogenic differentiation media were prepared following the instructions of *Human Mesenchymal Stem Cell Functional Identification Kit* (R&D Systems, Minneapolis, Minnesota, USA). Media were changed twice weekly in all the differentiation processes, and hDPSCs cultured without differentiation inducers were included as negative controls of differentiation. Finally, cultures were observed under an inverted light microscope (Leica DM IRB) and images were captured using a digital camera (Leica DFC300 FX, Leica Microsystems, Wetzlar Hesse, Germany).

##### 2.2.2.1 Osteogenic differentiation

Cells were seeded at 10,000 cells/cm^2^ in 48-well plates with the routine culture medium. When cultures reached 50%–70% confluence, medium was replaced with the differentiation medium. After 4 weeks, osteogenic differentiation was assessed by Alizarin Red staining. Briefly, cells were washed, fixed with 4% paraformaldehyde for 30 min, rinsed, and stained with 1.4% Alizarin Red (pH 4.1) (Merck KGaA) for 5 min.

##### 2.2.2.2 Adipogenic differentiation

For adipogenic differentiation, hPDLSCs were seeded on 48-well plates at a density of 15,000 cells/cm^2^ in the routine culture medium. After reaching 100% confluence, medium was replaced by the adipogenic differentiation medium. After 5 weeks, adipogenic differentiation was confirmed by the detection Fatty Acid-Binding Protein 4 (FABP4) (*Human Mesenchymal Stem Cell Functional Identification Kit*). Briefly, cells were fixed in 4% paraformaldehyde and permeabilized with Triton X-100. After blocking, the cultures were incubated with anti-FABP4, following the manufacturer’s indications. Finally, cells nuclei were counterstained with Hoechst 33,342.

##### 2.2.2.3 Chondrogenic differentiation

Chondrogenic differentiation was induced using the “pellet culture” technique described in the *Human Mesenchymal Stem Cell Functional Identification Kit*. In brief, 2.5 x 10^5^ cells were transferred into 15 mL conical tubes and centrifuged to form micromass pellets. Pellets were incubated with 0.5 mL of chondrogenic differentiation medium for 42 days, and then fixed in 4% paraformaldehyde, dehydrated in ethanol, cleared with xylene substitute, and finally embedded in paraffin. Chondrogenic differentiation was confirmed by Alcian Blue staining of 5 μm thickness sections followed by nuclear fast red counterstaining.

### 2.3 Obtaining of plasma rich in growth factors (PRGF) formulations

Two PRGF formulations were prepared: PRGF supernatant for culture medium supplementation, and PRGF membrane (mPRGF) for the central core of the bioengineered corneal grafts. Blood from two healthy donors was collected into tubes with 3.8% (wt/v) sodium citrate, after written informed consent was provided. Blood was then centrifuged at room temperature (RT) following manufacturer’s instructions (System V centrifuge; BTI Biotechnology Institute, Miñano, Álava, Spain). To obtain PRGF, the entire plasma column was obtained, ensuring the exclusion of the buffy coat. In order to prepare the culture medium supplement, the plasma was subsequently activated with calcium chloride according to the user’s guide for PRGF Endoret (BTI Biotechnology Institute) Following a 1 h incubation, the resulting PRGF clot was centrifuged at 1,000 g for 10 min at RT. The supernatant was then filtered, aliquoted, and stored at −80 °C. This PRGF supplement was utilized as a total replacement for FBS in all the differentiation processes to corneal phenotypes, when appropriate, and in the generation and maintenance of bioengineered constructs.

PRGF from two healthy donors was pooled to prepare mPRGFs for the grafts’ core. Fibrin membranes were prepared as previously described with some modifications (Fullaondo et al., 2025). For each membrane, 2 mL of PRGF was activated with calcium chloride and incubated at 37 °C for clot formation and retraction. The fibrin clot was then transferred to a fibrin membrane shaper (BTI Biotechnology Institute), and the resulting PRGF fibrin membrane was finally trimmed with a corneal trephine (blade 13.0 mm; BVI, Waltham, MA) to adjust its size.

### 2.4 hDPSCs differentiation to corneal phenotypes

The isolated hDPSCs were differentiated to cell phenotypes from the three corneal layers. Thus, for epithelial differentiation, subconfluent cell cultures were incubated with Supplemental Hormonal Epithelial Medium (SHEM) consisting in D-MEM/F-12, supplemented with 10 ng/mL epidermal growth factor (EGF) (Gibco-Thermo Fisher Scientific Inc.), 5 μg/mL insulin, 0.5% dimethyl sulfoxide (DMSO), 0.5 μg/mL hydrocortisone, 50 μg/mL gentamicin (the last four reagents from Sigma-Aldrich, Merck KGaA), and 5% PRGF supernatant for 14 days. In the case of corneal stromal differentiation, primary hDPSCs were maintained for 17 days in D-MEM/F-12 supplemented with 2 mM glutamine and 50 μg/mL gentamicin and including 1 mM 2-phospho-L-ascorbic acid, 10 ng/mL basic Fibroblast Growth Factor (bFGF), and 0.1 ng/mL Transforming Growth Factor beta 3 (TGF-β3) as keratocyte inductors (all cornea stromal inducers from Sigma-Aldrich, Merck KGaA). Furthermore, a preliminary dedifferentiation toward the neural lineage was a prerequisite for initiating the endothelial differentiation process. For this purpose, subconfluent hDPSCs cultures were maintained for 21 days in neurobasal A medium (Gibco-Thermo Fisher Scientific Inc.) supplemented with 50 μg/mL gentamicin, 20 ng/mL EGF, 40 ng/mL bFGF and one x B27 supplement (Gibco-Thermo Fisher Scientific Inc.). Subsequently, the neural differentiation medium was replaced by DMEM high glucose with glutamax including 1x penicillin-streptomycin solution (Gibco-Thermo Fisher Scientific Inc.) and 5% PRGF, and supplemented with 2 ng/mL bFGF, 2-mercaptoethanol, 10 ng/mL activin A (both from Gibco-Thermo Fisher Scientific Inc.), 10 ng/mL heregulin beta and 200 ng/mL Insulin Like Growth Factor (IGF-I) (both supplied by Sigma-Aldrich, Merck KGaA) as endothelial differentiation inducers. This last differentiation medium was maintained for 14 days. Media were changed twice weekly in all the differentiation processes.

To validate multilineage commitment, immunocytochemical analysis was performed to monitor changes in the antigenic expression profile of hDPSCs monolayer cultures. Lineage-specific differentiation was determined using antibodies against Pan-Cytokeratin (PanCK) (Sigma-Aldrich, Merck KGaA), Lumican (Lumi) (Abcam, Cambridge, UK) and Zonula Occludens-1 (ZO-1) (Invitrogen, Carlsbad, CA, USA) to identify epithelial, stromal, and endothelial phenotypes, respectively. For antigen detection, cells were fixed in either cold methanol (for PanCK) or 4% paraformaldehyde (PFA). PFA-fixed samples were subsequently permeabilized with 0.1% Triton X-100 at RT to detect Lumi and ZO-1. After blocking with 10% FBS in phosphate buffered saline (PBS), cells were incubated with primary antibodies overnight at 4 °C, followed by secondary antibody incubation for 1 h at RT, protected from light. Finally, cell nuclei were counterstained with Hoechst 33,342 (Molecular Probes- Thermo Fisher Scientific Inc.) for 5 min. Fluorescence images were captured using a digital camera (Leica DFC300 FX, Leica Microsystems, Wetzlar Hesse, Germany) coupled to an inverted fluorescence microscope (Leica DM IRB), maintaining identical imaging parameters for both non-differentiated and differentiated cell groups, and also for positive and negative controls. Quantitative analysis of the percentage of positive cells was conducted using ImageJ software (v1.54r).

### 2.5 Reverse transcription-quantitative polymerase chain reaction (RT-qPCR) analysis

For the quantification of relative gene expression levels, total RNA was isolated from both undifferentiated hDPSCs and cells subjected to corneal stromal differentiation. Human primary keratocytes (ScienCell Research Laboratories, Inc, Carlsbad, CA, USA) were included as positive control of *LUM* expression. Cells were cultured in 6-well plates, and RNA extraction was performed using the NucleoSpin RNA Plus kit (Macherey-Nagel, Düren, Germany) in accordance with the manufacturer’s protocol. Three biological replicates were included for each experimental condition. Briefly, cells were lysed and, after genomic DNA removal, RNA was trapped into the NucleoSpin columns, washed and eluted with RNase-free H_2_O by subsequent centrifugation steps.

RT-qPCR assays were designed and executed in strict accordance with the MIQE (Minimum Information for Publication of Quantitative Real-Time PCR Experiments guidelines) to ensure the reliability and reproducibility of the data (Bustin et al., 2009). One-step RT-qPCR was performed in a 10 μL reaction volume using the Reliance Multiplex RT-qPCR Supermix in combination with the *LUM* PrimePCR probe assay (both from Bio-Rad Laboratories Inc, Hercules, CA, USA). Based on evidence extracted from the scientific literature, Ribosomal protein, large, P0 (*RLP0*) and TATA-binding protein (*TBP*) (Bio-Rad Laboratories Inc.) were selected as internal reference genes for data normalization (Ferreira et al., 2024). All reactions were conducted on a CFX96 Touch Real-Time PCR Detection System (Bio-Rad Laboratories Inc). The thermal cycling profile consisted of an initial reverse transcription at 50 °C for 10 min, followed by polymerase activation and denaturation at 95 °C for 10 min, and 40 amplification cycles (95 °C for 10 s and 60 °C for 30 s). For each biological sample, a total of three technical replicates were included. Multiplexing was validated experimentally *via* comparative analysis of amplification efficiency and linearity against singleplex PCR reactions, as recommended by Broeders et al. (Broeders et al., 2014). RT-qPCR efficiency was assessed using the standard curve method using five-point, 10-fold serial dilutions (Svec et al., 2015). Relative gene expression was calculated using the geometric mean of the multiple reference genes for normalization, following the methods described by Vandesompele et al. (Vandesompele et al., 2002) and Hellemans et al. (Hellemans et al., 2007). *LUM* expression in human primary keratocytes served as the calibrator for ΔCt calculations. Statistical analyses were performed on log-transformed relative expression values with results presented as fold-change relative to the control group (Vandesompele et al., 2002; Livak and Schmittgen, 2001; Pfaffl, 2001).

### 2.6 Fabrication of complete corneal substitutes

The corneal grafts consisted of a mPRGF core with embedded cells resembling the stromal component, sandwiched by another cell layers. Two corneal substitutes were assessed depending on the inclusion of non-differentiated or differentiated hDPSCs. The fabrication process of the tri-layer graft expanded over 4 days in both cases. On day 1, undifferentiated hDPSCs or those previously differentiated toward corneal stromal phenotype were encapsulated within the mPRGF matrix. For this purpose, 2 mL PRGF were mixed with 50,000 hDPSCs and activated with calcium chloride, following the protocol previously described. These cellular mPRGFs were maintained ON under normal culture conditions with the routine culture medium in which FBS was replaced by PRGF (onwards, graft medium). Then, their size was adjusted to be placed in 48-well plates with a corneal trephine.

In parallel, hDPSCs were seeded on 48-well temperature-responsive plates (Nunc UpCell-Thermo Fisher Scientific Inc.) at a density of 10,000 cells/cm^2^ in graft medium and maintained under normal culture conditions. Half of the cell cultures were induced toward epithelial and endothelial phenotypes, while the remaining half continued to be maintained in graft medium. On the second day, cellularized mPRGFs were carefully placed onto super confluent cultures of hDPSCs (undifferentiated or epithelial committed) and then incubated at 20 °C to induce cell sheet detachment. After 1–2 h, the mPRGF, with the first cell sheet adhered to its underside, was transferred to a new well. This process was repeated sequentially to incorporate a second and a third cell sheet. Finally, the constructs were inverted (cell sheets facing upward) and transferred to permeable polyethylene terephthalate (PET) inserts (0.4 μm pore size) (Corning- Thermo Fisher Scientific Inc.). For differentiated constructs, epithelial and stromal differentiation media were placed in the upper and lower compartments, respectively. Graft medium was added in both insert compartments for grafts with undifferentiated hDPSCs. On the third day, Air-Liquid Interface (ALI) culture was initiated by removing the culture medium from the superior compartment. ALI culture was maintained for 24 h. To complete the bioengineered corneal graft, a new cell sheet of undifferentiated or endothelial differentiated hPDLSCs was adhered to the basal side of the construct, following the same detachment protocol previously described. All assays included at least three biological replicates of both cellular and acellular (control) grafts.

### 2.7 Optical and rheological properties of the bioengineered grafts

#### 2.7.1 Viscoelastic profiling

##### 2.7.1.1 Oscillatory Experiments

Oscillatory assays were conducted using a HAAKE MARS III rheometer (Thermo Fisher Scientific Inc.) equipped with parallel plates (20 mm, aluminum). Experiments were performed at 37 °C (following 2 min of thermal equilibration), maintaining a constant normal force between the plates (Fn = 0.1 N, auto-tension mode). Samples were covered with silicone oil to prevent dehydration. Amplitude strain sweeps were performed to determine the linear viscoelastic region (LVR) of the samples, applying an increasing strain (ϒ) ranging from 0.0001 to 10 at a constant frequency (f = 1 Hz).

##### 2.7.1.2 Frequency sweep tests

Frequency sweeps tests were conducted by applying a constant strain of 1% (ϒ = 0.01, within the LVR), with frequencies ranging from 0.1 to 10 Hz. The storage modulus (*G*′) and loss modulus (*G*″) were recorded as a function of frequency.

#### 2.7.2 Light transmittance

After washing grafts with PBS, they were trephined to a specific diameter to fit the surface area of a 96-well plate. The spectral absorbance of the samples in the 380–730 nm visible light range was measured using a multimode microplate reader (Synergy H1, Agilent, Santa Clara, CA, USA). Absorbance values (A) were then transformed into transmittance (T) using the following formula: T = 100*10^(−A)^.

### 2.8 Viability of hDPSCs in the grafts

Cell viability of the complete corneal substitutes was quantified using the WST (tetrazolium salt, 4-[3-(4-iodophenyl)-2-(4-nitrophenyl)-2H-5-tetrazolio]-1,3-benzene disulfonate) colorimetric assay (Sigma-Aldrich, Merck KGaA). Grafts were incubated with WST reagent at 37 °C for 1.5 h, following the manufacturer’s instructions. Absorbance at 450/620 nm was directly proportional to the number of living cells.

### 2.9 Stability and remodel of the corneal grafts

Bioengineered grafts were transferred to 48-well plates and maintained for 5 days in graft culture medium under normal culture conditions. Conditioned media recovered from the lower compartment of the inserts after collecting the graft samples, and from the 48-well plates at day 1 and at the final point time, was centrifuged and stored at −80 °C until assay. For measurement of the mPRGF fibrin degradation, D-dimer protein fragment was quantified using an ELISA kit (Thermo Fisher Scientific Inc.). In addition, procollagen type I (Abcam) was also determined for the remodel assays.

Furthermore, constructs stability was quantified in terms of mass preservation using the formula: Mass loss (%) = ((M_d1_-M_d5_)/M_d1_) * 100. For this purpose, grafts were weighed at the baseline, after 1 day and at the end point of the assay (5 days). Weight differences obtained by this method could be in consonance with the remodel process in the grafts.

### 2.10 Histological analysis

Bioengineered grafts both immediately post-fabrication and at the endpoint of the remodeling assay were fixed in neutral buffered formalin (Bio-Optica Milano Spa, Milan, Italy) for 24 h, dehydrated with graded ethanol, cleared in pure xylene, paraffin-embedded and sectioned into slices about 5 µm in thickness (FFPE sections) using a rotary microtome RM2255 (Leica Biosystems, Germany). Histological slices were stained with Harris Hematoxylin and alcoholic Eosin Y solution (Sigma-Aldrich, Merck KGaA) to evaluate the structural organization of the corneal graft, the cell distribution, and other morphological characteristics of the constructs developed. In addition, FFPE sections of the constructs at the endpoint of the remodel assay were analyzed for collagen type I deposition (Abcam). All immunohistochemistry was based on streptavidin-biotin complex with Vector-VIP peroxidase (Vector Laboratories Inc., Burlingame, CA, USA) and 10% v/v diluted Harris Hematoxylin as nuclear counterstain. Images were captured with a digital camera (Leica DFC300 FX, Leica Microsystems) coupled to a brightfield microscope (Leica DM LB).

### 2.11 Statistical analysis

Results were expressed as mean ± standard deviation. Shapiro-Wilk was performed to verify the parametric statistical assumptions of normality. For longitudinal multiple comparisons of the same graft across different time points, a mixed-model repeated-measures ANOVA was performed, assuming a Gaussian distribution and applying the Geisser-Greenhouse correction to account for violations of sphericity. Post-hoc analyses were conducted with Tukey’s adjustment. To compare grafts containing undifferentiated hDPSCs against those with differentiated cells, a two-tailed Student’s t-test was employed for normally distributed data with homogeneous variances. In cases where the assumption of homoscedasticity was violated, Welch’s correction was applied. For non-normally distributed populations, the non-parametric Mann-Whitney U test was utilized. All statistical analyses were performed using GraphPad Prism software (version 10.6.1; GraphPad Software, San Diego, CA, USA), being set the level of statistical significance as p < 0.05.

## 3 Results

### 3.1 Human dental pulp stem Cell characterization

**FIGURE 1:**
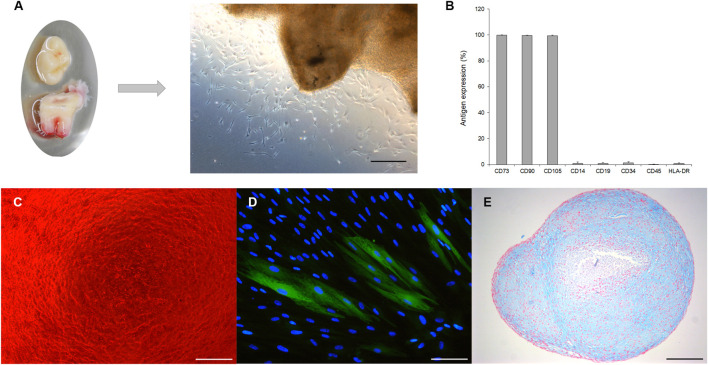
Isolation and characterization of hDPSCs **(A)** Impacted third molars were sectioned at the cementum-enamel junction to retrieve the dental pulp tissue, from which primary cultures of human dental pulp stem cells were established. **(B)** Quantification of the surface expression ofCD73, CD90, CD105, CD14, CD19, CD34, CD45 and HLA-DR, confirming the stemness of isolated hDPSCs. **(C)** Photomicrographs of osteogenic differentiated cells after alizarin red staining. **(D)** Detection of FABP4 after adipogenic differentiation (green staining). Nuclei were counterstained with Hoechst (blue). **(E)** Alcian blue staining of hDPSCs pellet sections after 42 days of chondrogenic differentiation. Scale bars = 400 μm **(A,C)**, 100 μm **(D)** and 200 μm **(E)**.

Primary hDPSCs, were isolated from the third molars following mechanical cleavage at the cementum-enamel junction. The resulting cultures displayed a characteristic fibroblast-like morphology (Figure 1A). Immunophenotypic characterization *via* flow cytometry confirmed the high expression of the mesenchymal markers CD73 (99.8% ± 0.2%), CD90 (99.7% ± 0.2%) and CD105 (99.5% ± 0.5%). Conversely, the cells were negative for hematopoietic and endothelial markers, including CD14 (0.9% ± 1.0%), CD19 (0.9% ± 0.6%), CD34 (1.2% ± 1.1%), CD45 (0.2% ± 0.1%) and HLA-DR (0.7% ± 0.5%) (Figure 1B). The multilineage potency was validated through successful differentiation into three mesodermal lineages. Osteogenic induction for 28 days resulted in the formation of a mineralized extracellular matrix, as evidenced by Alizarin Red S staining of calcium deposits (Figure 1C). Adipogenic potential was confirmed by the cytoplasmic expression of FABP4 (Figure 1D). Finally, chondrogenic capacity was demonstrated after 42 days of pellet culture, with Alcian Blue staining identifying the accumulation of sulphated glycosaminoglycans in the extracellular matrix (Figure 1E).

### 3.2 hDPSCs differentiation to corneal phenotypes

**FIGURE 2:**
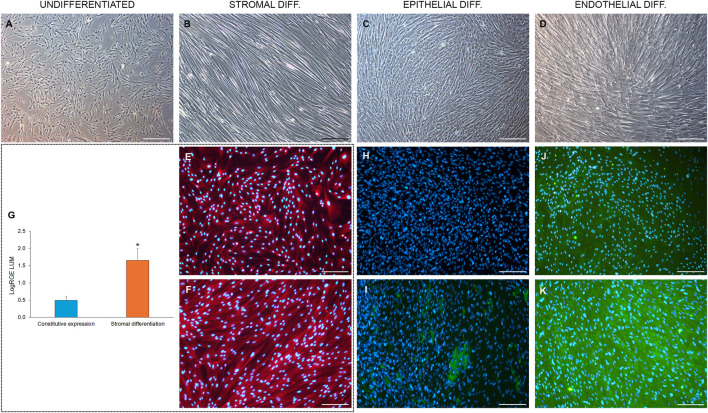
Morphological and immunophenotypic changes in hDPSC monolayer cultures after differentiation. **(A)** Established primary hDPSCs culture displaying a spindle shape morphology. **(B)** hDPSCs exhibiting a flattened morphology with long cytoplasmic processes following corneal stromal differentiation. **(C)** Transition to a cuboidal appearance of dental pulp stem cells upon epithelial differentiation. **(D)** hDPSCs beginning to adopt the characteristic hexagonal morphology of cornea endothelial cells following a sequential induction protocol consisting of 21 days in neural differentiation medium followed by 14 days of endothelial differentiation. **(E)** Baseline lumican expression (red fluorescence) in undifferentiated hDPSCs. **(F)** Lumican proteoglycan expression in dental pulp after 17 days of stromal differentiation. **(G)** Quantification by RT-qPCR of the *Lumican* (*LUM*) expression in hDPSCs before and after their induction toward the corneal stromal phenotype. *p = 0.0047 when compared to *LUM* expression in undifferentiated cells (95% CI). **(H)** Absence of constitutive PanCK expression in undifferentiated hDPSCs. **(I)** *De novo* expression of PanCK (green fluorescence) after 14 days of epithelial induction. **(J)**. Detection of constitutive ZO-1 expression (green fluorescence) in hDPSCs. **(K)** Increased expression of ZO-1 after endothelial differentiation. Images **(A–D)** were obtained under phase-contrast microscopy. **(E, F, H–K)** are fluorescence micrographs where cell nuclei were counterstained with Hoechst (blue fluorescence). Scale bars = 400 μm **(A,C,D)** and 200 μm **(B,E,F,H,I,J,K)**.

Two types of corneal grafts incorporating either undifferentiated or differentiated hDPSCs were developed. For the last bioengineered constructs, cells were induced to differentiate toward the specific cell phenotypes of the three corneal layers. Following induction, significant morphological alterations were observed. In the stromal lineage, cells became flattened or stellate, featuring long cytoplasmic processes (Figure 2B). Cells subjected to epithelial differentiation transitioned from their initial spindle-shaped morphology (Figure 2A) to a more cuboidal appearance (Figure 2C). Finally, cells induced toward an endothelial phenotype began to adopt a characteristic hexagonal morphology typical of the corneal endothelium (Figure 2D). To confirm the multilineage commitment, changes in the antigenic expression profile of hDPSCs following differentiation were analyzed. Thus, *de novo* expression of PanCK was observed exclusively in response to epithelial induction (Figures 2H,I), whereas a significant upregulation of ZO-1 was detected following differentiation toward endothelial corneal phenotype compared to baseline levels (Figures 2J,K, respectively). No significant differences in lumican expression were detected *via* immunocytochemical analysis between undifferentiated hDPSCs and those differentiated toward a corneal stromal phenotype, since this proteoglycan was detected constitutively in hDPSCs (Figures 2E,F). However, quantitative RT-qPCR analysis revealed a significant upregulation of *LUM* mRNA in hDPSCs following stromal induction (Figure 2G). The relative gene expression (logRGE) levels were significantly higher in the differentiated group compared to undifferentiated control (p < 0.005), indicating a robust transcriptional commitment toward the corneal keratocyte phenotype.

### 3.3 Optical and rheological properties of the bioengineered grafts

Strain sweeps were performed to determine the linear viscoelastic region of the samples; an increasing strain (ϒ) was applied (ranging from 0.0001 to 10) at a constant frequency (f = 1 Hz). The storage modulus (*G*′) remained constant, and thus independent of the strain, until reaching a critical value (ϒ = 0.1), which corresponded to the upper limit of the linear viscoelastic region. Consequently, a strain of 0.01 (1%) was selected for the subsequent frequency sweeps. In these tests, the frequency varied from 0.1 to 10 Hz, while the storage modulus (*G*′) and loss modulus (*G*″) were recorded as a function of frequency.

**FIGURE 3:**
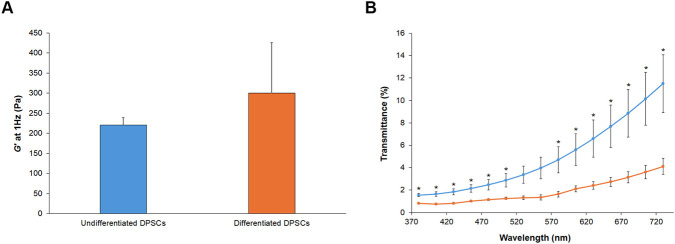
Rheological and optical characterization of the bioengineered corneal grafts. **(A)** Viscoelastic profiling of constructs containing either undifferentiated hDPSCs or hDPSCs differentiated toward cell phenotypes of the three corneal layers. The storage modulus (*G*′) was recorded as a function of frequency to assess structural stability. **(B)** Optical transmittance in the 380–730 nm visible light spectrum for the two constructs developed. In both plots, grafts containing undifferentiated and differentiated cells are represented in blue and orange, respectively. *Significance vs. differentiated DPSC group at the identical wavelength: p = 0.0013 (380 nm); p = 0.0017 (405 nm); p = 0.0026 (430 nm), p = 0.0050 (455 nm), p = 0.0077 (480 nm); p = 0.0102 (505 nm), p = 0.1000 (530 nm), p = 0.1000 (555 nm), p = 0.0114 (580 nm), p = 0.0132 (605 nm), p = 0.0124 (630 nm), p = 0.0115 (655 nm), p = 0.0104 (680 nm), p = 0.0097 (705 nm), p = 0.0088 (730 nm). (95% CI). (n = 4 and three biological replicates for rheological and optical characterization, respectively).

Both bioengineered constructs exhibited predominantly elastic behavior, with *G*′ consistently exceeding *G*'’ across the entire frequency range. For undifferentiated and differentiated hDPSC grafts, *G*′ vs. *G″* values were 221.00 ± 17.85 Pa vs. 39.81 ± 6.49 Pa and 299.91 ± 126.41 Pa vs. 41.44 ± 18.00 Pa, respectively, thus maintaining *G*''*/G*′ ratios below 0.25 across all conditions (data not shown). No significant differences regarding the storage modulus were found between the two graft types (p > 0.05) (Figure 3A).

Regarding optical properties, both constructs showed a wavelength-dependent increase in light transmission. Transmittance was lowest in the blue/violet region (∼380–450 nm) and increased progressively toward the red end of the spectrum (∼700+ nm). However, the incorporation of differentiated hDPSCs resulted in a statistically significant reduction in transparency across the visible spectrum compared to constructs containing undifferentiated cells (Figure 3B).

### 3.4 Viability of hDPSCs in the grafts

**FIGURE 4:**
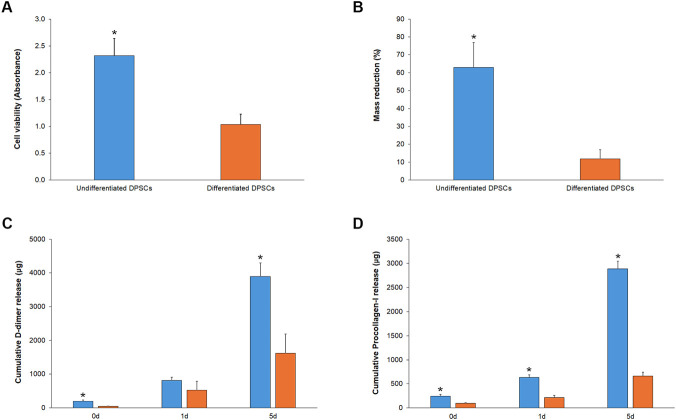
Stability and remodel of the corneal grafts. **(A)** Assessment of graft cellularity and post-fabrication cell viability. **(B)** Constructs stability was quantified in terms of mass preservation, with the plot illustrating weight differences between day 1 and day 5 post-assembly. **(C)** Graft degradation was assessed by measuring the release of D-dimer fragments from the mPRGF into the conditioned media after 5 days of culture. **(D)** To evaluate matrix remodeling, Procollagen Type I levels were quantified at the same experimental time points. In all plots, grafts containing undifferentiated and differentiated cells are represented in blue and orange, respectively. *Significance vs. differentiated DPSC group: **(A)** p = 0.0040; **(B)** p = 0.0146; **(C)** p = 0.0024 (day 0), p = 0.1000 (day 1), p = 0.0049 (day 5); **(D)** p = 0.0034 (day 0), p = 0.0005 (day 1), p < 0.0001 (day 5). (95% CI). All assays performed with n = 3 biological replicates.

Viability of the cellular component in both grafts was assessed by quantification of the cell metabolic activity, resulting in great significant differences in the absorbance values (2.32 ± 0.32 and 1.03 ± 0.2 absorbance units for grafts with undifferentiated and differentiated hDPSCs, respectively) (Figure 4A).

### 3.5 Stability and remodel of the corneal grafts

Constructs stability was quantified by assessing mass preservation between the first and fifth days of culture. The initial weight immediately following fabrication (day 0) was excluded from the analysis to account for the transition from air-liquid interface conditions to the fully submerged state required for the stability assay. A statistically significant reduction in graft mass was observed in constructs incorporating undifferentiated hDPSCs. Thus, grafts containing differentiated cells underwent a mass loss of 11.7% ± 5.2% after 5 days of incubation. In contrast, constructs combined with undifferentiated hDPSCs exhibited a five-fold greater reduction, losing 63.0% ± 13.9% of their initial mass (Figure 4B).

Regarding the remodel assays, the release of D-dimer fragments and procollagen type I into the conditioned media was quantified at day 1 and at the study endpoint. Statistically significant differences in the cumulative release of both proteins were observed between the two types of cellular scaffolds. As illustrated in Figure 4C, constructs incorporating undifferentiated hDPSCs released a significantly higher quantity of D-dimer after 5 days of incubation (3,892 ± 401 μg) compared to those with differentiated cells (1,618 ± 572 μg). Similarly, undifferentiated dental pulp stem cells in the corneal grafts synthesized a significantly greater amount of procollagen type I at all time points. Specifically, at the end of the culture period, the cumulative procollagen type I release reached 2,891 ± 156 μg for the undifferentiated hDPSCs, whereas only 664 ± 79 μg was released by cells differentiated toward corneal phenotypes (Figure 4D).

### 3.6 Histological analysis

**FIGURE 5:**
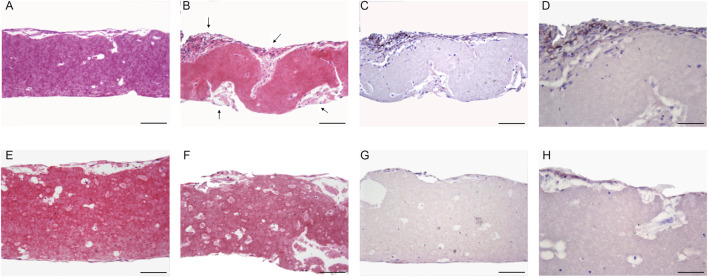
Histological evaluation of bioengineered corneal grafts. **(A,E)** Paraffin-embedded sections stained with Hematoxylin and Eosin of constructs incorporating undifferentiated hDPSCs and those differentiated toward corneal phenotypes, respectively, immediately post-fabrication. **(B,F)** Representative images of the same constructs following a 5-day stability assay; arrows indicate cellular masses infiltrating the mPRGF fibrin matrix. **(C,D,G,H)** Immunohistochemical analysis of type I collagen deposition (brown signal) at the assay endpoint. Panels **(C,D)** show grafts including undifferentiated cells at different magnifications, while **(G,H)** correspond to grafts incorporating differentiated hDPSCs. Scale bars = 100 μm **(A–C, E–G)** and 50 μm **(D,H)**. (n = 3 biological replicates).

Both bioengineered grafts containing either undifferentiated hDPSCs or those differentiated toward corneal cell phenotypes, were histologically evaluated immediately after their fabrication (Figures 5A,E, respectively) and at the end point of the stability assay. Hematoxylin-Eosin staining revealed profound structural alterations within the mPRGF matrix of grafts incorporating undifferentiated hDPSCs, where extensive cellular masses infiltrating the fibrin matrix were evidenced (Figure 5B). Such areas of active tissue remodeling were less prominent in sections of grafts containing differentiated cells (Figure 5F).

Immunohistochemical analysis for type I collagen further substantiated these findings, confirming different remodeling rates between the two corneal substitutes. Thus, the mPRGF fibrin was being extensively replaced through the active deposition of type I collagen by undifferentiated hDPSCs into a nascent matrix (Figures 5C,D). In contrast, matrix substitution was limited to small, superficial regions of the PRGF membrane when differentiated cells were incorporated into the bioengineered construct (Figures 5G,H).

## 4 Discussion

Many studies have examined the use of adult mesenchymal stem cells (MSCs) from several sources such as adipose tissue, the umbilical cord, and dental pulp, among others, for being included in regenerative therapies (Della Rocca et al., 2025; Hoang et al., 2022). MSCs are self-renewing, multipotent stem cells with the ability to differentiate into various cell phenotypes. Thus, MSCs from dental pulp have been considered for the cellular component of bioengineered corneal grafts in this research. hDPSCs have been isolated and immunophenotypically characterized to confirm their undifferentiated state and stemness. Moreover, they have been shown to differentiate to multiple mesodermic phenotypes such as osteoblast, chondrocyte and adipocyte lineages. This multilineage plasticity of hDPSCs has been widely described, suggesting a differentiation potential that resembles the versatility of pluripotent embryonic stem cells (Yang et al., 2018; Stefańska et al., 2024; Al Madhoun et al., 2021). Moreover, hDPSCs have emerged as an ideal candidate for cell-based therapies due to their minimally invasive procurement. These dental cells derived from the embryonic neural crest (NC) (He et al., 2025; Solis-Castro et al., 2022), a highly migratory and multipotent cell population. NC cells migrate extensively throughout the embryo, giving rise to diverse structures including the peripheral nervous system, the craniofacial skeleton and connective tissue, and specific cardiovascular components. Furthermore, they differentiate into a broad array of specialized cell types (Motohashi and Kunisada, 2026). Similarly, cell phenotypes constituting the three main corneal layers derived from the cranial ectoderm. Thus, the outermost surface of the cornea is covered by the stratified epithelium, a direct descendant of the ectoderm, while the middle stromal layer and the innermost corneal endothelium are both derived from neural crest cells (Williams and Bohnsack, 2020; Walker et al., 2020). Thus, the constitutive expression of lumican and ZO-1 in hDPSCs is biologically consistent, given the shared ontogeny between corneal stroma and endothelium and dental pulp. Notably, both markers were significantly upregulated following stromal and endothelial induction, respectively. Furthermore, successful epithelial differentiation was also achieved, as evidenced by the *de novo* expression of the PanCK marker. These findings underscore the inherent plasticity of hDPSCs and their capacity to adopt specific corneal phenotypes when cultured under appropriate inductive conditions.

Two distinct corneal graft models were developed utilizing a PRGF-derived fibrin membrane as a bioactive scaffold. In both constructs, dental pulp cells were embedded into the membrane mimicking the native corneal stroma. The first model incorporated undifferentiated hDPSCs into the three layers, whereas the second utilized cells differentiated toward corneal epithelial, stromal, and endothelial phenotypes. Both bioengineered constructs were maintained in a culture medium enriched with PRGF supernatant instead of FBS, to ensure a xeno-free environment during the fabrication process. Thus, avoiding the potential risks of disease transmission, xeno-immunization against bovine antigens, anaphylactic reactions, and ethical concerns (Duarte et al., 2023; Weber et al., 2025).

Rheological and optical properties of both corneal grafts were evaluated. In this sense, both bioengineered grafts exhibited predominantly elastic behavior, maintaining *G*''/*G*′ ratios below 0.25 across all conditions. *G*′ values of both constructs consisting of a mPRGF core are consistent with literature reports for fibrin-based tissue substitutes (Du et al., 2022; Namli et al., 2026; Borchers and Pieler, 2010), ensuring the necessary mechanical support for surgical handling. Regarding optical properties, grafts incorporating undifferentiated hDPSCs exhibited significantly higher transmittance levels compared to their differentiated counterparts. The main objective of these bioengineered constructs is to promote the healing of damaged corneal structures in order to restore tissue functionality, acting as a transient scaffold rather than a permanent replacement. Consequently, these initial transparency levels are expected to evolve as the engineered fibrin-based graft undergoes progressive post-transplantation remodeling by cellular components. This transition typically involves a gradual thinning of the provisional scaffold, coupled with an enhancement in optical clarity as the fibrin is replaced by a more organized, secondary extracellular matrix (González-Gallardo et al., 2023; Sanz-Horta et al., 2023; Gandhi et al., 2020). Certainly, these expectations were confirmed through the stability assay in which both bioengineered grafts were maintained at 37 °C for 5 days. Mass of the engineered constructs with undifferentiated hDPSCs was diminished by more than 60% while a slightly more than 10% reduction was seen when differentiated cells were included. This reduction in the mass of the bioengineered grafts would be associated with the active scaffold remodeling process. Specifically, as the initial fibrin matrix undergoes enzymatic degradation, it is progressively replaced by *de novo* collagen synthesized by undifferentiated hDPSCs. This precise synchronization of graft degradation kinetic and the rate of new tissue formation would ensure structural integrity maintenance throughout the regeneration process. Moreover, this progression from transient fibrin support to a cell-derived extracellular matrix is a hallmark of functional tissue maturation (Mayorca-Guiliani et al., 2025; Abolhasani et al., 2025).

While the initial cell counts to fabricate the bioengineered grafts were comparable between both corneal substitutes, a more than two-fold increase in metabolic activity was detected in constructs incorporating undifferentiated hDPSCs. A plausible explanation for these findings could be based on the superior proliferative capacity of undifferentiated cells, since the widely described ongoing competition during the G1 phase of the cell cycle between differentiation and continued self-renewal (Zhao et al., 2020; Victor et al., 2020). Undifferentiated hDPSCs likely underwent significant proliferation through the 4-day fabrication period, leading to a substantial increase in cell density that correlates with the higher metabolic activity observed in these constructs. Alternatively, the PRGF-fibrin microenvironment may trigger specific modulations in the mitochondrial activity of undifferentiated hDPSCs. In this regard, mitochondria, traditionally viewed as cellular powerhouses, are increasingly recognized as dynamic regulators of cell identity, metabolic reprogramming, and stress responsiveness (Labusca and Zara-Danceanu, 2025; Fu et al., 2025; Zhang et al., 2025; Main et al., 2023). A diminished cell activity would be associated with a less capacity of graft remodel, as evidenced by the results of D-dimer and procollagen type I release. Undifferentiated hDPSCs demonstrated an enhanced capacity to actively remodel the fibrin core, whereas a more limited remodeling potential was observed in cells that had undergone differentiation. The fibrin matrix was progressively replaced by type I collagen, the primary structural component of the corneal stroma, although the remodeling kinetics differed significantly between the two bioengineered corneal substitutes. Furthermore, bioactive signals within the mPRGF-fibrin matrix are likely released in a gradual manner, guiding the regeneration of damaged corneal tissues (Anitua et al., 2013). In conclusion, the remodeling of fibrin-based bioengineered constructs comprising a PRGF membrane core integrated with hDPSCs could be tailored by controlling the differentiation status of the incorporated cells, increasing their versatility for tissue engineering applications.

In recent years, several biomaterial strategies have emerged as promising grafts for corneal regeneration, each aiming to mimic the native tissue’s structural integrity and function. Among them, collagen-based scaffolds offer excellent biocompatibility and optical clarity; however, they frequently suffer from poor mechanical stiffness and accelerated enzymatic degradation *in vivo*, often requiring chemical crosslinking that can compromise cytocompatibility (Huang et al., 2026). To overcome these mechanical constraints, photocrosslinkable hydrogels like GelMA have gained traction due to their tunable physical properties and printability. Nevertheless, GelMA grafts carry inherent risks of ultraviolet or blue-light toxicity during polymerization, and their synthetic modification can hinder natural cellular remodeling (Ahearne et al., 2020). On the other hand, decellularized corneal matrices provide the most accurate anatomical blueprint by preserving innate extracellular matrix cues. Yet, their clinical translation is heavily bottlenecked by substantial batch-to-batch variability, complex processing protocols, and the potential retention of immunogenic detergents or nucleases (Li and Wang, 2025). Alternatively, synthetic polymers deliver highly predictable mechanical profiles and structural stability, but their lack of intrinsic cell-signaling domains often results in poor epithelialization, chronic inflammation, or graft integration failure (Visalli et al., 2024).

To bridge these clinical and technical gaps, bioengineered grafts based on PRGF technology present a highly advantageous, autologous alternative. Unlike synthetic polymers or modified hydrogels, mPRGF is intrinsically non-immunogenic, entirely bypassing the risk of disease transmission or foreign-body responses. Furthermore, mPRGF inherently contains a physiological cocktail of essential morphogens and growth factors that actively drive cellular recruitment and matrix maturation, circumventing the mechanical fragility of pure collagen and the processing complexities of decellularized matrices. Crucially, mPRGF exhibits remarkable cellular compatibility and structural versatility; it can be efficiently combined with distinct corneal cell phenotypes to closely recapitulate the anatomical complexity of either partial-thickness (lamellar) or full-thickness (penetrating) corneal grafts. Consequently, mPRGF positions itself as a dynamically active, patient-specific platform capable of orchestrating true and targeted corneal regeneration.

Initially designed to replicate the cornea’s trilayered cellular architecture, these bioengineered grafts represent a promising biomimetic candidate for future clinical applications in transplantation surgery. Furthermore, the remarkable plasticity of hDPSCs, coupled with the proven efficacy of PRGF across diverse medical fields, suggests that this methodological platform could be readily adapted for a wide range of other tissue engineering applications. While these *in vitro* findings are highly encouraging, future research is required to validate the *in vivo* behavior of these corneal grafts combining the PRGF technology and human dental pulp stem cells.

## Data Availability

The raw data supporting the conclusions of this article will be made available by the authors, without undue reservation.
